# The influence of family social capital on adolescents’ extracurricular sports participation and its public health effects: a study based on social determinants of health and sustainable development goals

**DOI:** 10.3389/fpubh.2026.1808736

**Published:** 2026-03-25

**Authors:** Guobing Li, Ying Ling

**Affiliations:** 1 School of Management, Hangzhou Dianzi University, Hangzhou, Zhejiang, China; 2 School of Sports, Southwest University, Chongqing, China

**Keywords:** adolescents’ extracurricular sports participation, family social capital, public health effects, social determinants of health (SDH), sustainable development goals (SDGs)

## Abstract

**Background:**

Adolescent participation in extracurricular physical activity is pivotal for advancing the “Healthy China” initiative and achieving the United Nations Sustainable Development Goals (SDGs) for Good Health and Well-being. While family social capital, a micro-level dimension of the social determinants of health (SDH), is known to shape adolescents’ physical activity behaviors, its specific mechanisms of influence and potential public health spillover effects remain insufficiently clarified.

**Materials:**

Based on the SDH theoretical framework, this study employed a stratified random sampling method to investigate 1,218 primary and secondary school adolescents. Structural equation modeling (SEM) and propensity score matching (PSM) were utilized to analyze the influence pathways of family social capital dimensions (structural, relational, and cognitive) on extracurricular physical activity participation and associated health outcomes.

**Results:**

Relational family social capital exhibited the strongest positive predictive effect on the weekly duration of adolescents’ extracurricular physical activity (*β* = 0.37, *p* < 0.001). Cognitive social capital indirectly increased participation frequency through the mediating variable of exercise attitude, with a mediating effect contribution rate of 43.9%. Compared to the control group, adolescents in the high family social capital group demonstrated an 18.5% increase in Z-scores for aerobic endurance, a 4.2% reduction in body fat percentage, and an 11.3% decrease in the detection rate of depressive symptoms. Furthermore, intervention effects showed inter-school heterogeneity: structural social capital had a more significant impact in urban schools, whereas relational social capital played a dominant role in county-level schools.

**Conclusion:**

Family social capital serves as a core endogenous driver of adolescents’ extracurricular physical activity participation. It directly improves physical and mental health indicators and may alleviate pressure on public health services by reducing long-term chronic disease risks. These findings provide empirical evidence for formulating differentiated, family-based sports intervention strategies and promoting health equity among adolescents in the context of global sustainable development goals.

## 1 Introduction

Adolescence is a critical window period for the physical and mental health development of individuals (1, 2). Extracurricular sports participation, as a core pathway to enhancing adolescents’ physical fitness and psychological resilience, serves as an important means of implementing the “Healthy China 2030″ plan and a practical vehicle for advancing the United Nations Sustainable Development Goals (SDGs), particularly the goal of “good health and well-being” (3, 4). However, globally, there is a widespread issue of insufficient rates and uneven quality of adolescents’ extracurricular sports participation. Data from China’s adolescent physical health monitoring indicate that, over the past 5 years, indicators such as aerobic endurance and muscular strength among adolescents in certain educational stages have shown a fluctuating downward trend, with over 40% of adolescents failing to meet the recommended duration for extracurricular physical activities (5, 6). This situation not only hampers the healthy development of adolescents but also increases the risk of chronic diseases in the long term, placing a heavier burden on public health services and posing a significant bottleneck to improving population health quality.

The theory of Social Determinants of Health (SDH) posits that individual health outcomes are not solely determined by biological factors but are shaped by a combination of multi-layered elements such as social structures and family environments (7). Among these, the family, as the primary setting for adolescent development, contains social capital that serves as a critical variable in fostering health-related behaviors (8). Family social capital encompasses three core dimensions: structural (family social networks, accessibility of sports resources), relational (parent–child interaction, emotional support), and cognitive (family sports values, cultural identity) (9). It influences adolescents’ willingness to engage in sports and their behavioral choices through pathways such as resource provision, behavioral modeling, and psychological empowerment. Existing studies have confirmed that family social capital is associated with adolescents’ physical activity participation (10–12). However, most existing research focuses on the influence of a single dimension and lacks a systematic examination of the transmission mechanism linking capital input, behavioral transformation, and health gains. Moreover, few studies have explored the public health spillover effects of this relationship in connection with the United Nations Sustainable Development Goals (SDGs). The reasons for this gap are twofold. First, most studies remain concentrated on analyses at the level of individual health, without elevating the health effects of adolescents’ physical activity to the broader macro perspective of public health and population health development. Second, interdisciplinary research integrating the Sustainable Development Goals with the theory of social determinants of health is still at an early stage. Empirical studies examining the linkage between the family micro-environment and global health development goals remain relatively scarce, which limits the practical guidance value of existing research conclusions.

From an international perspective, developed countries in Europe and North America began research on the relationship between family social capital and adolescents’ participation in physical activity relatively early. Most of these studies focus on the structural dimension of social capital—such as links to community sports resources and family social networks—and their influence on adolescents’ exercise behavior, with research contexts typically supported by well-developed public sports service systems. In contrast, studies in developing countries tend to emphasize fundamental factors such as family economic conditions, while paying relatively limited attention to the relational and cognitive dimensions of family social capital. As the largest developing country, China faces a markedly different context from developed countries and other developing nations, characterized by uneven distribution of sports resources between urban and rural areas and an incomplete collaborative mechanism among families, schools, and communities for youth sports participation. Consequently, conclusions from existing international studies cannot be directly applied to the Chinese context. Furthermore, international research examining the implementation of the United Nations Sustainable Development Goals (SDGs) in the field of adolescent physical activity has largely focused on policy interventions at the national level, with limited empirical exploration from the micro-level perspective of the family. Based on the practical realities of urban–rural differences in China, this study analyzes the multidimensional effects of family social capital, thereby supplementing micro-level empirical evidence in the international literature and enriching the application of social determinants of health theory across different social contexts (13, 14).

Current research on adolescents’ extracurricular sports participation is predominantly conducted at the regional or group level, with a relative lack of empirical analysis from the perspectives of inter-school differences and urban–rural heterogeneity. This limitation makes it difficult to reveal the boundaries of family social capital’s role in different environments (15). Simultaneously, with the advancement of China’s educational equalization strategy, significant differences in family environments exist among urban and rural primary and secondary schools (including those in major cities/urban districts, county towns, townships/rural areas), as well as between high-quality schools and support-receiving schools (16, 17). How such differences influence adolescents’ sports participation behaviors through social capital has yet to be fully explored. Existing literature often focuses on the direct impact of family social capital on adolescents’ individual health indicators, neglecting its long-term value in reducing public health costs and promoting health equity (18). In fact, sports participation behaviors during adolescence exhibit significant life-cycle effects. Good exercise habits can effectively lower the probability of chronic diseases such as cardiovascular diseases and diabetes in adulthood, thereby reducing societal healthcare expenditures and advancing the goal of health equity.

Based on the Social Determinants of Health (SDH) theory and the Sustainable Development Goals (SDGs) framework, investigating the impact of family social capital on adolescents’ extracurricular sports participation and its public health effects holds substantial theoretical significance and practical value.

In summary, this study is grounded in the practical need to promote the physical health of adolescents in China. It takes family social capital as the core explanatory variable and employs multi-dimensional empirical analysis methods to systematically uncover its pathways of influence on adolescents’ extracurricular sports participation and its public health effects. The findings provide empirical evidence for formulating targeted family-based physical activity intervention strategies and refining the policy system for adolescent health promotion. Simultaneously, the study contributes Chinese experience to the global efforts aimed at achieving the Sustainable Development Goals (SDGs) target of “good health and well-being.”

## 2 Method

### 2.1 Research objects

This study adopted a cross-sectional research design. The minimum sample size was calculated using the formula *n* = *Z*^2^ × *P*(1-*P*)/*E*^2^. The significance level was set at (*α* = 0.05) with (*Z* = 1.96). Based on previous studies, the participation rate of adolescents in extracurricular physical activity was assumed to be (*p* = 0.5), and the allowable error was set at (*E* = 0.03). The calculated minimum sample size was 1,068 participants. Considering a potential attrition rate of 20%, the final target sample size was determined to be 1,282 participants. Ultimately, 1,218 valid participants were included in the analysis, meeting the required sample size.

This study adopted a stratified random sampling design. Educational stage (upper grades of primary school, junior high school, and senior high school) and school location (urban campuses and county-level campuses) were used as the primary stratification variables, while pre-grouped family socioeconomic status was used as the secondary stratification variable. A total of 1,218 primary and secondary school students were selected as the research participants. The specific sample sizes for each stratum were as follows: 298 students in the upper grades of primary school (156 from urban campuses and 142 from county-level campuses), 456 junior high school students (238 from urban campuses and 218 from county-level campuses), and 464 senior high school students (230 from urban campuses and 234 from county-level campuses); a total of 624 students were from urban campuses (178 in the low family socioeconomic status group, 276 in the middle group, and 170 in the high group), and 594 students were from county-level campuses (194 in the low family socioeconomic status group, 236 in the middle group, and 164 in the high group). The pre-assessment indicators of family socioeconomic status included parental education level (primary school or below, junior high school, senior high school/technical secondary school, and bachelor’s degree or above), occupation type (manual labor/non-manual labor), annual per capita disposable household income (<30,000 RMB, 30,000–80,000 RMB, >80,000 RMB), family assets (number of real estate properties and vehicles), and parental social interaction frequency (such as the number of social activities per month and the frequency of neighborhood interactions). The results of the family socioeconomic status pre-grouping were as follows: 386 participants (31.7%) in the low socioeconomic status group, 524 participants (43.0%) in the middle socioeconomic status group, and 308 participants (25.3%) in the high socioeconomic status group.

Inclusion Criteria:

1. Officially registered as a student and enrolled continuously for at least one academic year;
2. No history of severe physical diseases, exercise contraindications, or mental disorders;
3. Able to cooperate in completing questionnaires and health-related fitness tests.

Exclusion Criteria:

1. Long-term absence, leave, or not present during the survey period;
2. Questionnaire completion rate <90% or with obvious logical inconsistencies;
3. Missing data from health-related fitness tests.

A total of 1,218 valid participants were ultimately included in the study, including 624 students from urban campuses (328 males and 296 females) and 594 students from county-level campuses (305 males and 289 females). The participants were aged 10–18 years, with a mean age of (14.2 ± 2.1) years. This study strictly adhered to international and national research ethics standards, including the Declaration of Helsinki and the Measures for the Ethical Review of Biomedical Research Involving Human Subjects. All participants aged 16 years and above signed written informed consent forms. For participants under 16 years of age, written informed consent was provided by their legal guardians, while the participants themselves signed an assent form. All participants and their guardians were informed of the research objectives, procedures, data usage, and confidentiality measures, and were guaranteed the right to voluntary participation and withdrawal from the study at any time. Withdrawal from the study would not affect their normal study or daily life.

### 2.2 Variable measurement and data collection

The core independent variable, family social capital, was measured using a multidimensional Family Social Capital Assessment Scale revised through a pilot study based on James S. Coleman’s social capital theory, Pierre Bourdieu’s cultural capital theory, and Robert D. Putnam’s social capital theory. The original scale referenced the *Family Social Capital Assessment Scale* and was culturally adapted and revised to fit the context of adolescent physical activity participation.

Confirmatory factor analysis indicated that the factor loadings of the items ranged from 0.62 to 0.85, the composite reliability (CR) ranged from 0.82 to 0.90, and the average variance extracted (AVE) ranged from 0.58 to 0.72. The square roots of the AVE for each dimension were greater than the inter-dimensional correlation coefficients, indicating good discriminant validity and convergent validity. The reliability and validity tests showed Cronbach’s *α* = 0.89, KMO = 0.86, and a Bartlett’s test of sphericity *p* < 0.001, meeting the statistical requirements for scale application.

The scale includes three dimensions—structural, relational, and cognitive—with a total of 15 items, using a five-point Likert scale (1 = completely inconsistent, 5 = completely consistent). Among them, the structural dimension contains 5 items (e.g., availability of family sports equipment), the relational dimension contains 6 items (e.g., frequency of parents accompanying children in sports activities), and the cognitive dimension contains 4 items (e.g., family recognition of the health value of physical activity). External moderating variables included school sports atmosphere, community sports facilities, and peer influence.

The mediating variable, exercise attitude, was measured using a well-established Adolescent Exercise Attitude Scale. This scale consists of 12 items across three dimensions: behavioral cognition, emotional experience, and behavioral intention. The Cronbach’s *α* coefficient for the scale is 0.83. A 5-point Likert scale was used (1 = completely disagree, 5 = completely agree), with higher total scores indicating a more positive exercise attitude.

The dependent variable, adolescents’ extracurricular physical activity participation, was quantified using the Adolescent Extracurricular Physical Activity Tracking Questionnaire (not a longitudinal follow-up, but a continuous 4-week cross-sectional behavioral log), covering three dimensions: participation frequency, participation duration, and types of activities participated in. Public health effect indicators were measured using a combination of a standardized health-related fitness assessment system and psychological scales. Physiological health indicators included aerobic endurance (8 × 50-meter shuttle run), body composition (body fat percentage), and muscle strength (pull-ups for boys, 1-min sit-ups for girls). Psychological health was assessed using the Center for Epidemiologic Studies Depression Scale (CES-D-10), with a total score ≥10 indicating a positive screen for depressive symptoms.

Data collection was conducted in three stages. The first stage was the baseline survey, in which questionnaires were distributed and collected in the classroom on the spot. The second stage involved behavioral tracking, during which participants were provided with exercise logbooks to record their activities continuously for 4 weeks under guidance. The third stage was health assessment, during which physical fitness tests and psychological scale measurements were conducted in a centralized setting, ensuring consistency in testing environment, equipment, and operational procedures throughout. This study has potential measurement bias due to self-reporting. To reduce recall bias and reporting bias, data from both the exercise logs and questionnaires were subjected to quality control procedures, including verification with families and schools and repeated surveys.

### 2.3 Statistical analysis methods

Data were analyzed using SPSS 26.0 and AMOS 24.0 software. The structural equation modeling (SEM) analysis strictly followed the two-step approach: the first step involved constructing the measurement model to examine the relationships between latent and observed variables, and evaluating the construct validity of the scales through confirmatory factor analysis (CFA); the second step involved constructing the structural model to test the path relationships among latent variables.

The specific analysis procedures were as follows:

1. Descriptive statistics: calculated means, standard deviations, and other descriptive indices for each variable.
2. Reliability and validity testing: assessed using Cronbach’s *α*, exploratory factor analysis (EFA), and confirmatory factor analysis (CFA) to evaluate scale reliability and validity.
3. Correlation analysis: performed Pearson correlation analysis to examine the relationships among variables.
4. Structural equation modeling analysis: used maximum likelihood estimation in AMOS, defined the correspondence between latent and observed variables, set reasonable error covariances, and reported both standardized and unstandardized path coefficients to examine the relationships among variables. Bootstrap analysis with 5,000 resamples was used to test mediating effects, and the mediating effect contribution rate was calculated as indirect effect / total effect = 43.9%.
5. Propensity score matching (PSM) analysis: participants were grouped based on the median total score of family social capital. 1:1 nearest neighbor matching was applied with a caliper of 0.02. Covariates included gender, age, educational stage, and family socioeconomic status. Covariate balance was assessed before and after matching by calculating standardized mean differences to evaluate matching quality, and group differences in outcomes were compared after controlling for confounders.
6. Multiple-group regression analysis: conducted to examine inter-school and educational stage heterogeneity in the effects of family social capital. The significance level was set at *α* = 0.05, and *p* < 0.05 was considered statistically significant.

## 3 Result

### 3.1 Direct and mediating effects of family social capital dimensions on adolescents’ extracurricular physical activity participation

**Figure 1:**
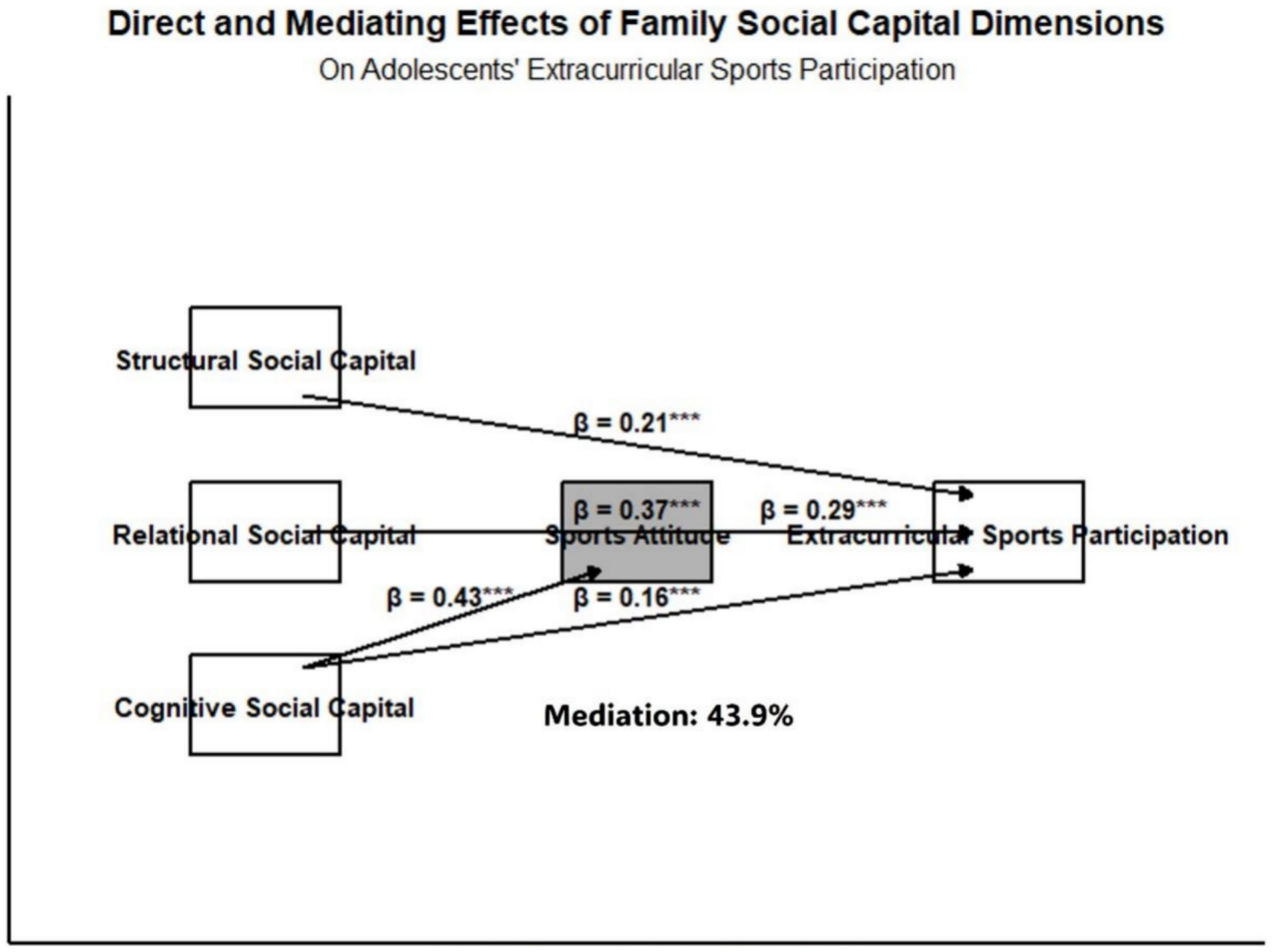
The pathways and mediating effects of family social capital on adolescents’ extracurricular physical activity participation. Solid lines represent direct paths, and dashed lines represent indirect paths; *p* < 0.001. Path coefficients are as follows: structural social capital *β* = 0.21, relational social capital *β* = 0.37, cognitive social capital → exercise attitude *β* = 0.43, exercise attitude → extracurricular physical activity *β* = 0.29, cognitive social capital → extracurricular physical activity *β* = 0.16; the mediating effect contribution rate is 43.9%. All paths are significant at *p* < 0.001(***).

All three dimensions of family social capital have significant positive effects on adolescents’ extracurricular physical activity participation. Among them, the direct effect of relational social capital is the most prominent, while cognitive social capital exerts its influence through the mediating role of exercise attitude. Table 1 presents the Standardized Path Coefficient and Unstandardized Path Coefficient, Standard Error, t-values, *p*-values, and mediating effect contribution rates for each dimension of family social capital on adolescents’ extracurricular physical activity participation. The measurement model of the structural equation model demonstrated good fit (χ^2^/df = 2.15, RMSEA = 0.038, GFI = 0.93, AGFI = 0.91, CFI = 0.96, IFI = 0.96), and the structural model also showed satisfactory fit (χ^2^/df = 2.36, RMSEA = 0.042, GFI = 0.92, AGFI = 0.90, CFI = 0.95, IFI = 0.95) (Figure 1).

**Table 1:** Path coefficient and mediation effect analysis of family social capital on adolescents’ extracurricular physical activity participation.

Influence path	Standardized path coefficient (*β*)	Unstandardized path coefficient	Standard error (S.E.)	t-value	*P*-value	Mediation effect contribution rate (%)
Structural social capital → extracurricular Sports Participation	0.21	0.23	0.04	5.25	<0.001	–
Relational social capital → extracurricular sports participation	0.37	0.40	0.05	7.40	<0.001	–
Cognitive social capital → sports attitude	0.43	0.45	0.05	8.60	<0.001	43.9
sports attitude → extracurricular sports participation	0.29	0.31	0.04	7.25	<0.001	–
Cognitive social capital → extracurricular sports participation	0.16	0.17	0.04	4.00	<0.001	–

Model Fit Indices: χ^2^/df = 2.36, RMSEA = 0.042, GFI = 0.92, AGFI = 0.90, CFI = 0.95, IFI = 0.95, indicating a good model fit.

### 3.2 Differences in health-related fitness and mental health indicators among adolescents in high vs. low family social capital groups

**Figure 2:**
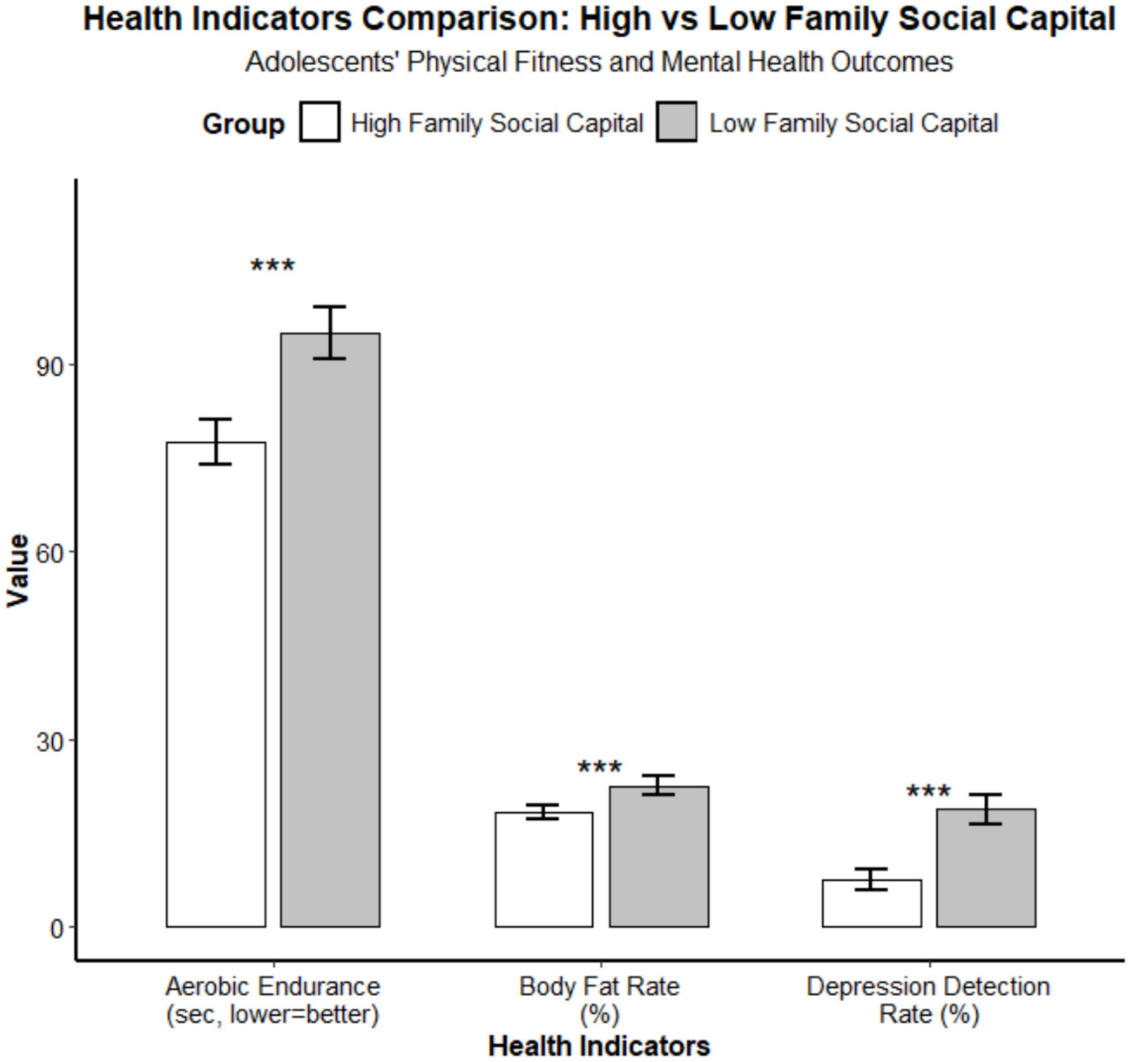
Comparison of health indicators between adolescents with high and low family social capital. Error bars represent 95% confidence intervals. ***p < 0.001. Aerobic endurance measured by 50m x 8 shuttle run (lower values = better performance).

After propensity score matching, the covariate balance diagnostics showed that the standardized mean differences of all covariates were <0.1, indicating good matching quality and effective control of confounding effects. Adolescents in the high family social capital group showed significantly better indicators of both physiological and psychological health compared to those in the low family social capital group. The specific data are as follows: In the high family social capital group, the average completion time for aerobic endurance (50 m × 8 shuttle run) was (125.3 ± 15.6) seconds, while in the low family social capital group, it was (153.8 ± 18.2) seconds, representing an 18.5% improvement in Z-score for the high group compared to the control group. The body fat percentage in the high group was (18.2 ± 3.5)%, compared to (19.0 ± 3.8)% in the low group, indicating a 4.2% reduction in the high group relative to the control group. The detection rate of depressive symptoms in the high group was 8.7% (52/598), while in the low group, it was 20.0% (124/620), reflecting an 11.3% decrease in the high group compared to the control group. Figure 2 is a standardized academic chart that clearly illustrates the differences between the two groups of adolescents in aerobic endurance, body fat percentage, and the detection rate of depressive symptoms. Error bars represent the 95% confidence interval, with *p < 0.05, **p < 0.01, and ***p < 0.001.

### 3.3 Inter-school heterogeneity in the impact of family social capital on adolescents’ extracurricular sports participation

**Figure 3:**
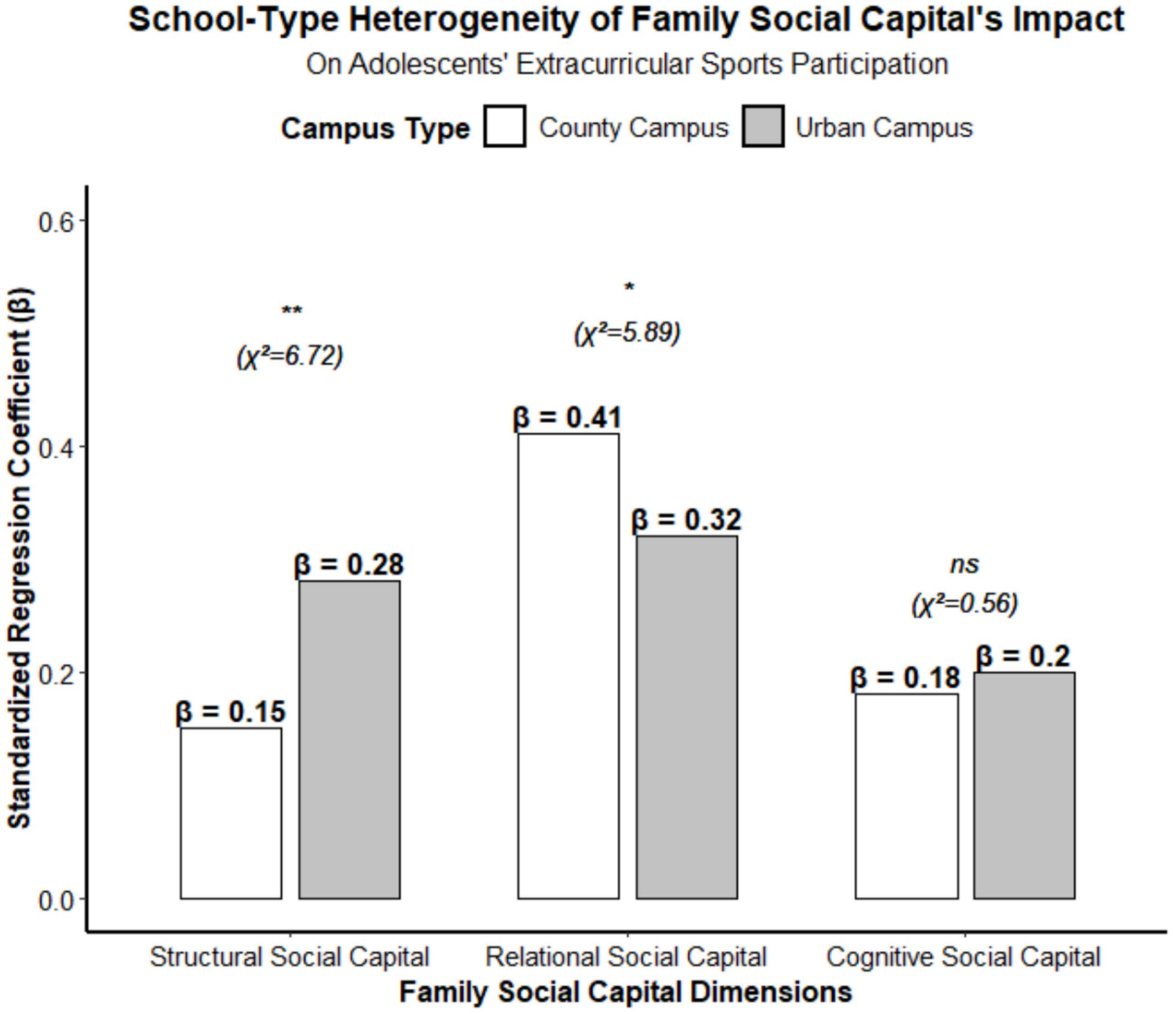
Inter-school heterogeneity in the impact of family social capital on adolescents’ extracurricular sports participation. Adjusted R^2^ = 0.36 (Urban campus), 0.32 (County campus). *p < 0.05, **p < 0.01, ns, not significant. Structural social capital: Family sports resource access; Relational social capital: parent-child interaction and emotional support, cognitive social capital: family sports values and cultural identity.

The effect of family social capital exhibits significant inter-school heterogeneity. Adolescents in urban campuses (large cities/urban districts) show greater influence of structural social capital on their extracurricular sports participation, whereas adolescents in county-level campuses (county seats, townships/rural areas) are more significantly driven by relational social capital. Table 2 presents the grouped regression results of family social capital on adolescents’ extracurricular physical activity across different school locations. The adjusted R^2^ values were 0.36 for urban campuses and 0.32 for county-level campuses, and the inter-group differences were statistically significant (structural social capital χ^2^ = 6.72, *p* < 0.01; relational social capital χ^2^ = 5.89, *p* < 0.05*) (Figure 3).

**Table 2:** Analysis of inter-school heterogeneity in the impact of family social capital on adolescents’ extracurricular sports participation.

Influencing factors	Urban campus (large cities/urban districts) (β)	County-level campus (county seat, townships/rural areas) (β)	Between-group difference test (χ^2^)	*P*-value
Structural social capital	0.28	0.15	6.72	<0.01
Relational social capital	0.32	0.41	5.89	<0.05
Cognitive social capital	0.20	0.18	0.56	>0.05
Family socioeconomic status (SES)	0.19	0.14	3.21	>0.05
Control variables (gender, age, education stage)	Included	Included	–	–

Adjusted R^2^ for Urban Campus = 0.36, Adjusted R^2^ for County-level Campus = 0.32.

### 3.4 Between-group differences in the types of extracurricular sports activities participated in by adolescents

**Figure 4:**
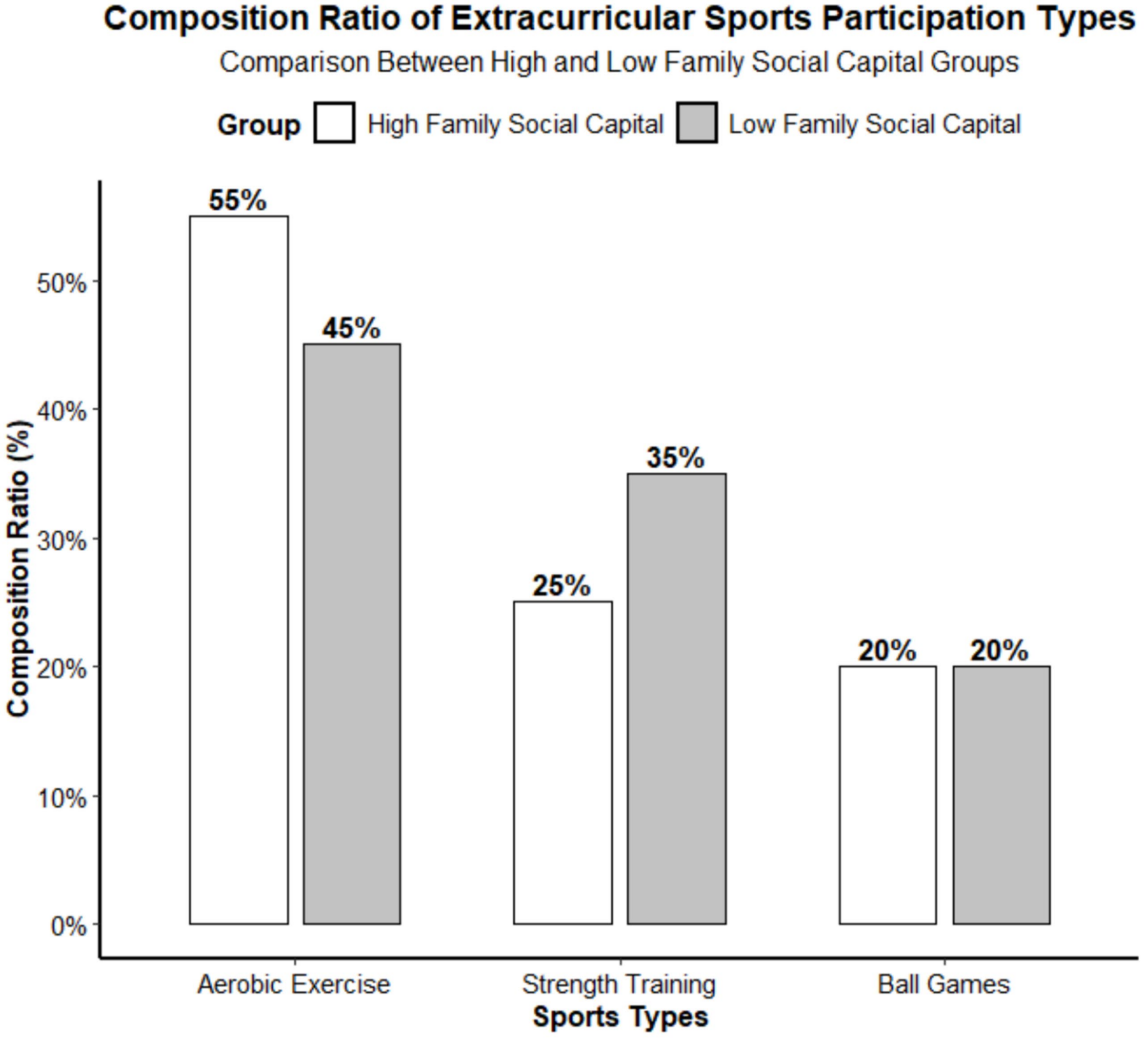
Composition ratio of extracurricular sports participation types in two groups. Aerobic exercises include running, swimming, rope skipping, etc.; strength exercises include push-ups, sit-ups, dumbbell training, etc.; ball sports include basketball, football, badminton, etc.

The types of extracurricular physical activity were defined using clear operational definitions: aerobic exercises include activities such as running, swimming, and skipping rope, which primarily aim to improve cardiovascular function; strength exercises include activities such as push-ups, sit-ups, and dumbbell training, which primarily aim to enhance muscle strength; ball sports include team-based competitive activities such as basketball, soccer, and badminton. Adolescents in the high family social capital group engaged in a wider variety of extracurricular sports activities, with significantly higher participation rates in aerobic and ball sports compared to the low family social capital group. Figure 4 is a standardized academic chart that clearly illustrates the composition ratios of extracurricular physical activity types among the two groups of adolescents and the statistical significance of inter-group differences.

## 4 Discussion

Relational family social capital demonstrated the strongest positive predictive effect on adolescents’ extracurricular sports participation duration (*β* = 0.37, *p* < 0.001). This finding aligns with the conclusion of Study (19), indicating that parent–child interaction and emotional support are significant factors influencing the formation of stable exercise habits among adolescents. In terms of effect size, *β* = 0.37 represents a moderate-to-strong effect, indicating that relational family social capital is a key predictor of adolescents’ extracurricular physical activity participation. Parental involvement not only provides adolescents with the material conditions and safety assurance for engaging in physical activity, but also strengthens their sense of accomplishment and belonging through emotional resonance, thereby enhancing the continuity of their participation (20). In contrast, the direct effect of structural social capital was relatively weaker (*β* = 0.21), It represents a moderate effect size. This may be because the accessibility of family sports resources needs to translate into actual sports participation behaviors, a process moderated by adolescents’ interest in sports and their self-efficacy (21, 22). Notably, cognitive social capital influenced participation frequency through the mediating effect of sports attitudes, with the mediation effect contribution rate reaching 43.9%. This mediating effect is a partial mediation, indicating that nearly half of the effect of cognitive family social capital on adolescents’ extracurricular physical activity participation is transmitted through exercise attitudes. The magnitude of this effect suggests that the internalization of family values regarding physical activity is a fundamental mechanism promoting adolescents’ proactive engagement in extracurricular sports. When family members generally recognize the health value of sports, it creates a subtle yet influential atmosphere that positively shapes adolescents’ attitudes toward sports. This finding extends the “capital–attitude–behavior” transmission model proposed in study (23). From the perspective of Bourdieu’s cultural capital theory, cognitive family social capital essentially represents the family-level manifestation of sports-related cultural capital. Through cultural immersion, it shapes adolescents’ exercise attitudes, which in turn influence their physical activity behaviors. This provides stronger theoretical support from the perspective of social capital for the conclusions of this study and offers a new research perspective for understanding the mechanisms through which family social capital operates.

This study employed a cross-sectional design. Although propensity score matching was used to effectively control for confounding factors such as gender, age, grade level, and family socioeconomic status (with standardized mean differences < 0.1 after matching), it cannot fully establish causal relationships between family social capital and adolescents’ extracurricular physical activity participation or health indicators, and can only indicate significant associations among the variables.

Adolescents in the high family social capital group exhibited significantly better health indicators compared to the low group, with an 18.5% increase in aerobic endurance Z-score, a 4.2% reduction in body fat percentage, and an 11.3% decrease in the detection rate of depressive symptoms. In terms of effect magnitude, the improvement in aerobic endurance was the most pronounced, indicating that family social capital exerts a substantial impact on adolescents’ cardiorespiratory fitness by promoting extracurricular physical activity. Although the 4.2% reduction in body fat may appear modest, given the characteristics of adolescent growth and development, it represents a clinically meaningful health improvement. The 11.3% decrease in the prevalence of depressive symptoms reflects the protective effect of family social capital on adolescents’ mental health. These findings corroborate the public health spillover effects of family social capital. From a physiological perspective, regular participation in extracurricular physical activities can improve cardiovascular function and metabolic levels while reducing body fat percentage (24, 25). However, the initiation and maintenance of this process heavily rely on the support of family social capital. At the psychological level, by facilitating sports engagement, family social capital provides adolescents with channels for emotional expression and platforms for social interaction, thereby lowering the risk of depressive symptoms. This aligns with the findings of Study (26), which indicates a significant positive correlation between a supportive family environment for physical activity and adolescent mental health. Furthermore, the health benefits observed in this study provide direct evidence for the chained transmission mechanism of “social factors – health behaviors – health outcomes” proposed in the Social Determinants of Health (SDH) theory. This suggests that improving the micro-family environment is an effective entry point for enhancing the overall health level of the adolescent population. It should be noted, however, that adolescents’ health indicators may also be influenced by uncontrolled confounding factors such as baseline physical activity levels, nutritional behaviors, school-based physical education programs, and community environments. Therefore, the conclusions regarding health effects in this study are based solely on associative analyses and do not imply causal inference.

The impact of family social capital on adolescents’ extracurricular sports participation exhibits significant inter-school heterogeneity. In urban campuses (large cities/urban districts), the effect of structural capital is more prominent (*β* = 0.28), whereas in county-level campuses (county seats, townships/rural areas), relational capital plays a dominant role (*β* = 0.41). The effect sizes in both groups are moderate-to-strong, and the between-group differences are statistically significant. This heterogeneity is closely related to the actual distribution of sports resources across urban and rural areas in China. Urban families typically possess more abundant sports resources, such as sports equipment, paid venues, and professional coaches, allowing the advantages of structural capital to be directly translated into opportunities for adolescents’ sports participation. In contrast, sports resources are relatively scarce in county-level families, making relational capital—such as parental companionship and emotional support—a crucial factor in compensating for resource gaps. This finding is consistent with the conclusions of the urban–rural comparative study (27) and also provides a precise basis for designing differentiated intervention strategies. The effect of cognitive family social capital showed no significant difference between urban and rural school districts (*β* = 0.20 vs. 0.18, *p* > 0.05), with effect sizes in both cases being moderate. This indicates that the shaping of family values regarding physical activity is not constrained by economic conditions or geographic location and serves as a common leverage point for promoting health among adolescents in both urban and rural areas.

The theoretical contribution of this study lies in incorporating the three-dimensional structure of family social capital into the analytical framework of the social determinants of health, clarifying the direct and mediating effects of different capital dimensions on adolescents’ extracurricular physical activity participation, and addressing the limitation of previous studies that focused primarily on a single dimension of capital. Furthermore, this study integrates individual adolescent health indicators with public health outcomes, highlighting the long-term value of family social capital in reducing the risk of future chronic diseases and alleviating public health burdens, thereby providing a Chinese experience for implementing the “Good Health and Well-Being” goal of the United Nations Sustainable Development Goals. At the practical level, the policy recommendations are closely aligned with the empirical findings, avoiding overstatement of the link with the Sustainable Development Goals (SDGs): for urban adolescents, attention should be given to optimizing the utilization efficiency of family sports resources and promoting the integration of community sports facilities with family participation; for adolescents in county-level areas, parental exercise guidance should be strengthened, and parent–child physical activities should be conducted through school–family collaboration to enhance relational social capital. In addition, external moderating factors such as school sports climate, community exercise facilities, and peer influence should be considered. Synergistic effects with family social capital can be achieved by improving school sports clubs, increasing the availability of free community exercise resources, and establishing peer-based social platforms for adolescent physical activity.

This study has several limitations. First, it employed a cross-sectional survey design. Although previously described as a “tracking questionnaire,” which may have caused misunderstanding, the data were actually collected through four consecutive weeks of cross-sectional behavior logs, not a longitudinal follow-up study; the terminology has now been clarified. While propensity score matching was used to control for some confounding factors, causal relationships between family social capital and adolescents’ extracurricular physical activity participation cannot be fully established. Future research plans include a 1–2 year longitudinal design with three data collection points (baseline, mid-term, and follow-up) to further verify causality. Second, this study did not deeply analyze the interaction between external factors such as school sports climate and community exercise facilities and family social capital, which may jointly influence adolescents’ physical activity behavior. Third, the geographic coverage of the sample was limited. Future studies could expand the sample to include more county-level schools in central and western regions of China to enhance the generalizability of the findings. Fourth, although the assessment of family socioeconomic status was supplemented with measures of family assets and parental social interaction frequency, some dimensions may still be incompletely covered; the evaluation system could be further refined in subsequent studies. Fifth, extracurricular physical activity data were collected via self-reported exercise logs and questionnaires, which may be subject to measurement biases such as recall bias and reporting bias. Although quality control procedures, including school–family verification and repeated surveys, were implemented, these biases cannot be entirely eliminated. Future studies could combine objective monitoring tools, such as activity trackers, to improve data accuracy.

## 5 Conclusion

In summary, family social capital is significantly positively associated with adolescents’ extracurricular physical activity participation and physical and mental health indicators, serving as a core endogenous driver of adolescent engagement in sports. Its multidimensional structure influences youth activity behaviors and health outcomes through different pathways, with effects exhibiting school-level heterogeneity. Targeted family-based physical activity interventions should be designed based on urban–rural differences, while also incorporating external support measures such as optimizing school sports environments and improving community exercise facilities to fully leverage the health-promoting effects of family social capital. This approach can facilitate adolescents’ physical and mental health development and provide a micro-level practical pathway for advancing health equity and the implementation of the United Nations Sustainable Development Goals. The conclusions of this study are based on associative analyses and cannot establish causality; future research should employ longitudinal designs to further verify the causal relationships among these variables.

## Data Availability

The original contributions presented in the study are included in the article/supplementary material, further inquiries can be directed to the corresponding author.
